# Developing an automatic treatment record review system for quality assurance of patient treatment delivery in radiation therapy

**DOI:** 10.1186/s13014-024-02582-8

**Published:** 2025-01-15

**Authors:** Peng Huang, Yingjie Xu, Fukui Huan, Yanxin Zhang, Min Ma, Kuo Men, Jianrong Dai

**Affiliations:** Department of Radiation Oncology, National Cancer Center/National Clinical Research Center for Cancer/Cancer Hospital, Chinese Academy of Medical Sciences and Peking Union Medical College, Beijing, 100021 China

**Keywords:** Treatment record, Plan delivery, Radiation therapy, Review, Anomaly

## Abstract

**Background and purpose:**

Treatment record contains most of information related to treatment plan delivery in radiation therapy. Reviewing treatment record is an important quality assurance (QA) task for safety and quality of patient treatments. This task is usually performed by senior medical physicists. However, it is time-consuming, tedious, and error-prone. To assist this process, a treatment record review system (TRRS) is developed to automatically review items related to treatment delivery record.

**Methods:**

The treatment record is firstly extracted from oncology information system (OIS). Based on the daily patient treatment information, the original plan from the treatment planning system is identified. Then the original plan and the delivered plan are correlated. Eight review categories (parameter consistency, treatment completeness, treatment progression, image guidance, override, treatment couch, documentation, and treatment mode) are created. Tailored rules are designed for various review items to automate the review process. As a result, for each daily treatment record, a reviewed flag (pass, failure, warning, and N/A) is assigned by the TRRS. Finally, this system is evaluated by 6 months patient treatment records collected in our institute and compared to the manual process on the same data.

**Results:**

TRRS processed a total of 76,651 treatment fractions from 4230 patients with an average of 574 treatments per day. The percentage of the detected anomalies among the total records was 0.76%. The average processing time was 3.9 s and 282 s per treatment record for the automatic and manual processes, respectively. Comparing with the manual process, the time efficiency of TRRS is improved by a factor of 72. The average numbers of anomalies detected by the automatic and manual processes are 21 and 13 per day, respectively. TRRS detects 61.5% more anomalies than those of the manual process.

**Conclusion:**

TRRS is not only efficient in processing a large amount of treatment records on a daily basis but also effective in finding more anomalies than those of physics weekly check. The application of the TRRS could significantly reduce the workload of the review physicists and let them focus on more important works related to patient safety.

## Introduction

Radiotherapy has been an effective way for treating cancer and has rapidly evolved in the past decades. Many new radiotherapy treatment techniques, such as intensity-modulated radiation therapy (IMRT), volumetric modulated arc therapy (VMAT), and stereotactic body radiation therapy (SBRT), are capable of delivering high-precision dose to tumors while safeguarding the surrounding health tissues. However, a small mistake in one step of the whole treatment process would result in serious problem at the subsequent steps [1–4]. Therefore, a careful inspection of daily treatment accuracy of the radiotherapy plans is necessary. Shafiq et al. presented a survey on international radiotherapy incidents. They found 19% of 3125 incidents happened in the treatment stage [5]. Ezzell et al. analyzed 173 problematic events and found 43% events happened in the treatment stage [6].

Treatment record review is a comprehensive inspection of various data associated with a patient's treatment, including the plan, delivery, patient setup and monitoring phases [7]. Eric et al. showed that weekly review of treatment record by a physicist could effectively reduce the occurrence of radiotherapy accidents. It was one of the most effective measures to ensure the quality control of patient treatment, with an effectiveness rate of more than 40% [8]. American Association of Physicists in Medicine (AAPM) Task Group (TG) 275 report and Medical Physics Practice Guideline (MPPG) 11.a further emphasized the importance of treatment record review in radiation therapy [9, 10]. Both reports recommended that a Qualified Medical Physicist (QMP) should perform treatment record review at least weekly and document it. In brief, treatment record review plays a crucial role in ensuring the accuracy, quality, and safety of radiation therapy treatments.

Manual review of treatment records is a time-consuming process, especially when dealing with a large amount of complex treatment plans [9]. It requires significant human resources, including the time and expertise of qualified personnel such as medical physicists. In hospitals where staffing is limited, allocating physicists for treatment record reviews is difficult. Given the complexity of treatment plans, only relying on manual method may increase the risk of missing critical details. Physicists conducting manual reviews may also apply individual criteria. It could lead to inconsistencies between reviewers, affecting the reliability and uniformity of the quality assurance program. In addition, Manual review processes is mentally demanding. Repetitive work would lead to fatigue, which may affect the attention to detail and thoroughness of the review, potentially increasing the risk of oversights [11, 12].

Several researchers developed methods to assist manual review process with computer-aided solutions [13–18]. Holdsworth et al. developed an in-house software called Verifier, which was designed to improve the efficacy and efficiency of radiation therapy treatment planning and quality control review [19]. Yang et al. introduced the development and implementation of a framework to automate the quality control (QC) step in radiotherapy treatment plan verification [20]. Currently, studies on automatic treatment record review are rare. Xia et al. developed an automated system called CATERS (Computer Aided Treatment Event Recognition System) to analyze electronic treatment records and detect treatment events in radiation therapy. The system improved the efficiency of treatment monitoring by automating the search for deviations from the physician's intention [21].

The physics group in our institute developed a treatment plan review system (TPRS), also called Automatic review (AutoReview), which improved the efficiency of review by nearly 60 times and increased the anomaly detection rate by 19.2% [22]. Based on the TPRS and the recommendation of AAPM TG275 report, we further developed an automatic treatment record review system (TRRS). TRRS was built upon TPRS and integrates with the MOSAIQ Version 2.80 (Elekta Medical Systems, USA). It is expected that this system could improve the reliability and efficiency of current treatment record review process, and help physicians, physicists and therapists quickly and accurately find errors and potential risks that may occur during the treatment process.

## Methods and materials

### System architecture

**Fig. 1:**
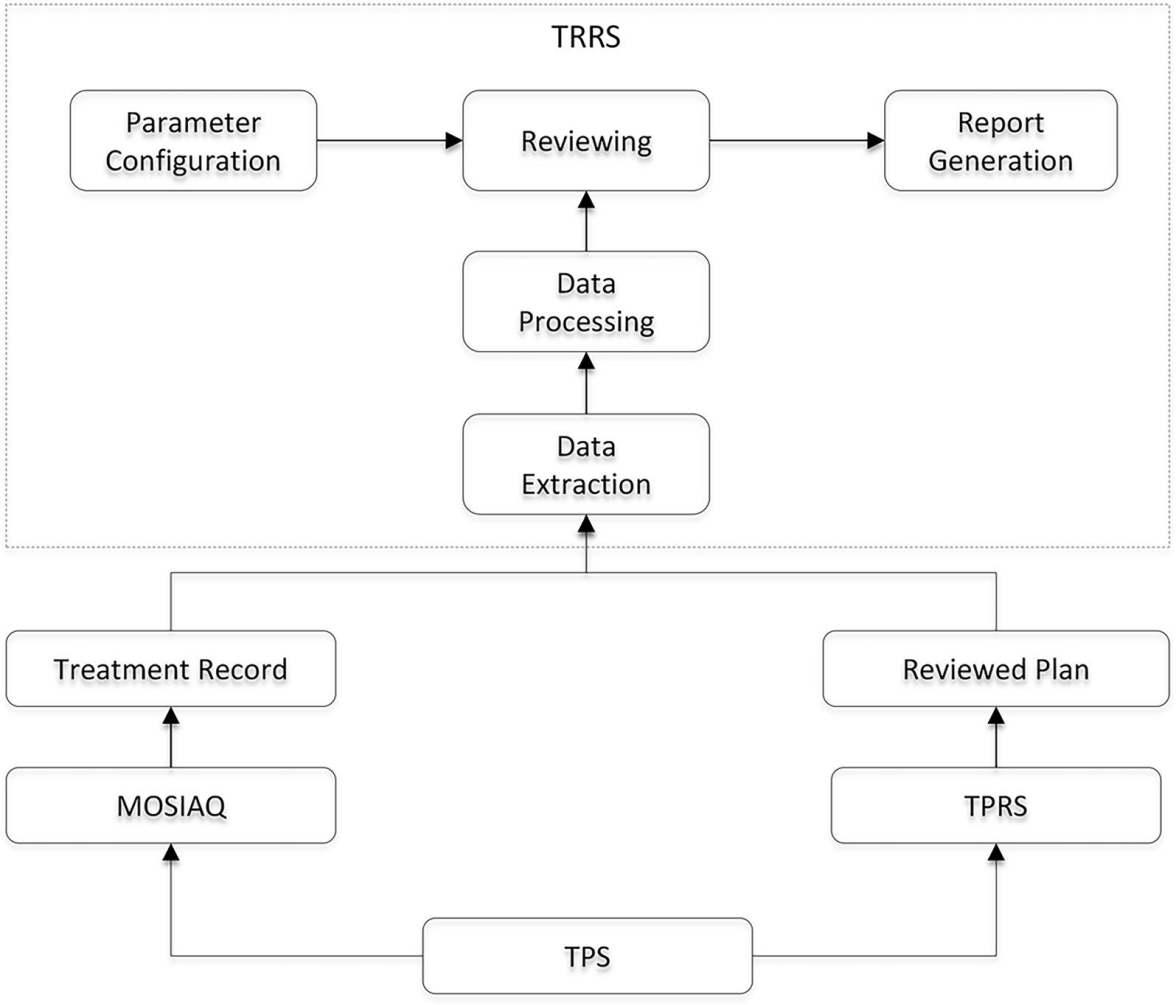
The architecture of TRRS

The system architecture of TRRS follows the B/S (Browser/Server) model, utilizing Java and HTML languages for programming. The primary program server operates on the Windows 2016 platform, with MySQL serving as the database management system. This architecture enables review physicists to access TRRS from any workstation within the hospital LAN via a standard web browser, which facilitates the display and analysis of review results. It consists of five main components, data extraction, data processing, the automated review program, parameter configuration, and report generation, as shown in Fig. 1.

### Data acquisition

The data used by TRRS is mainly structured data obtained from two sources as shown in Fig. 1. The original treatment plan is first generated in Treatment Planning System (TPS) and then transferred to MOSIAQ for treatment delivery. After delivery, the treatment record is generated and stored in MOSIAQ. The structured data of the delivered plan was extracted from MOSAIQ and sent to TRRS for further analysis. The original treatment plan is also transferred to TPRS for physics review. After review, the structured data of the reviewed plan is extracted from the TPRS and sent to TRRS for further analysis. Once both delivered and reviewed plan data are obtained, a one-to-one correspondence between them is established by matching key field. Specifically, the Prescription Unique Identifier (SIT_ID) within the MOSAIQ database is used in this task.

### Review items

The review items are categorized into eight groups: parameter consistency, treatment completeness, treatment progression, image guidance, override, treatment couch, documentation, and treatment mode. The descriptions of these items are listed in Table 1. The review status of a treatment record falls into one of four cases:

1. Pass: the value of this item is normal.
2. Failure: the value of this item is incorrect.
3. Warning: the value of this item is questionable and needs further manual review.
4. N/A: the value of this item is no applicable.

**Table 1:** The details of the review items

Categories	Details
Parameter consistency	Consistency between treatment delivery parameters and treatment plan parameters, which included scrutiny of machine specifications, modality, energy, beam type, source-to-surface distance (SSD), segment count, monitor units (MU), gantry angle, collimator settings, couch angle, jaw configuration, and multi-leaf collimator (MLC) positions, etc
Treatment completeness	Delivery of all treatment fields in the plan
	Delivered MU did not exceed planned MU
Treatment progression	Cumulative dose and remaining dose
	Accuracy of the remaining session
	Consistency between the daily treatment dose and the prescribed dose
	Treatment calendar that has been postponed for an extended period or discontinued altogether
	Dose verification prior to stereotactic treatment
Image guidance	Image approval in accordance with departmental policies
	Applied shifts
	Isocenter on the CBCT(cone-beam computed tomography) matched plan
	Selection of IGRT (image-guided radiation therapy) scan template and parameters meet clinical requirements
	IGRT frequency adherence to medical directives
	IGRT registration deviation did not exceed predefined threshold
Overrides	Override records, including instances such as couch position exceeding tolerance, inconsistent field parameters, abnormal dose tracking, treatment fractionation mode not consistent with the prescription, and any other deviations
Treatment couch	Discrepancies between the treatment couch position (vertical, lateral, longitudinal, and rotational) and the reference couch position
Documentation	Completeness of treatment-related documentation
	Approval of documents by both the planning physicist and review physicist
Treatment mode	Treatments completed out of clinical mode
	The individual performing the QA model treatment

### System design

**Fig. 2:**
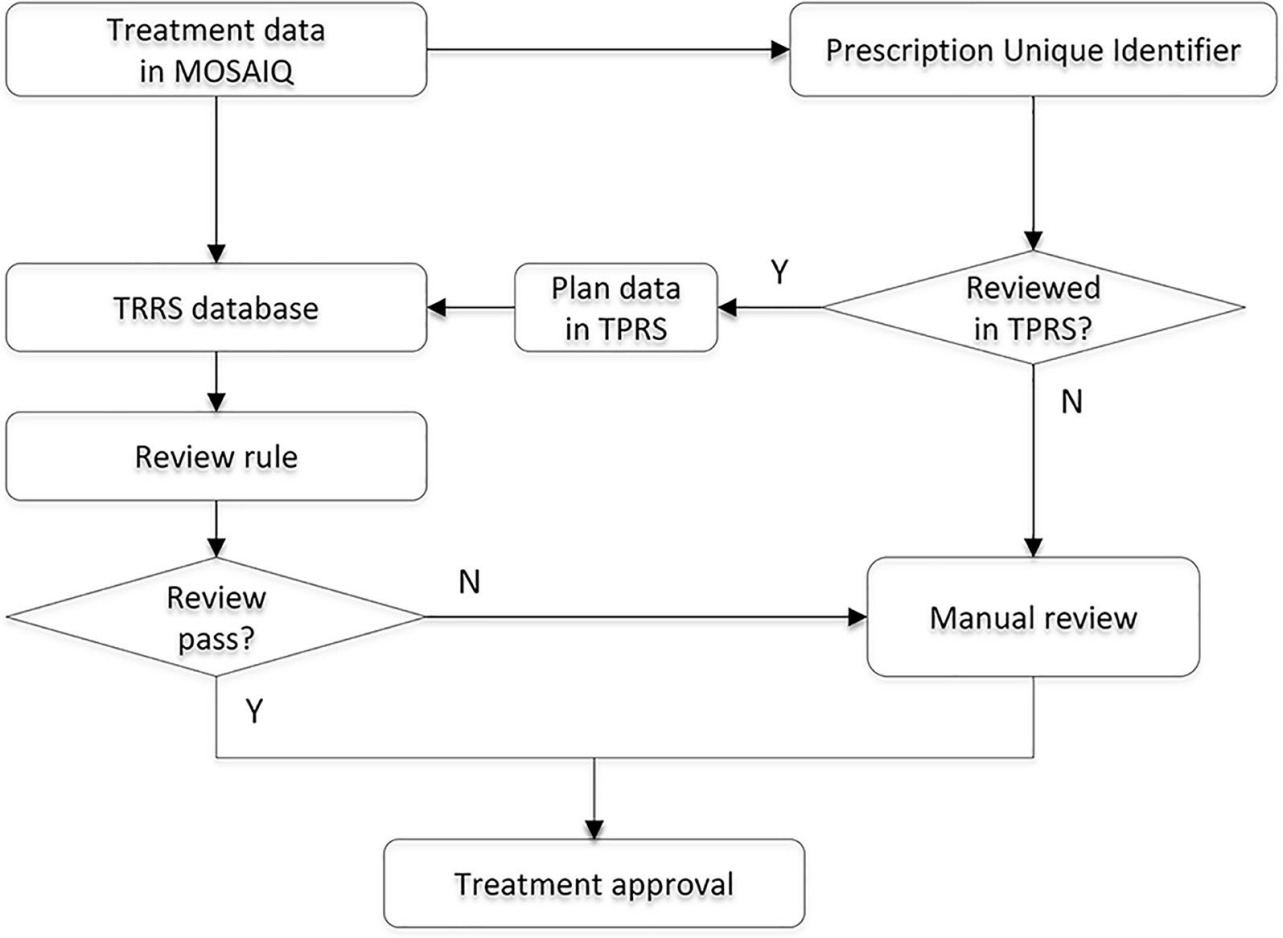
Workflow of TRRS

The workflow of TRRS is illustrated in Fig. 2. The core automatic review program resides on the server. It systematically retrieves treatment records obtained from the MOSAIQ system on a daily basis. These records contain data such as prescriptions, iso-centers, treatment fields, positioning fields, treatment couch, and images. With patient prescription information, TRRS seamlessly matches and retrieves corresponding original treatment plan obtained from TPRS. Tailored rules are created for various review items to automate the review process. Upon completion, TRRS generates a detailed report for each review process. Subsequently, the review physicists focus primarily on TRRS review results, manually scrutinize and address any anomalous items highlighted in TRRS.

### System evaluation

The treatment records over a period of 6 months from August 2023 to January 2024 were collected in our institute. Two senior physicists manually reviewed these treatment records meanwhile these data were also processed by TRRS. A comparative analysis was conducted between the manual review results and those processed by TRRS. The statistical analysis was performed using SPSS 21.0 software and χ^2^ test was employed to evaluate the consistency between the TRRS and manual review results. A significance level of *P* < 0.05 was established, with any disparities considered statistically significant.

## Results

**Fig. 3:**
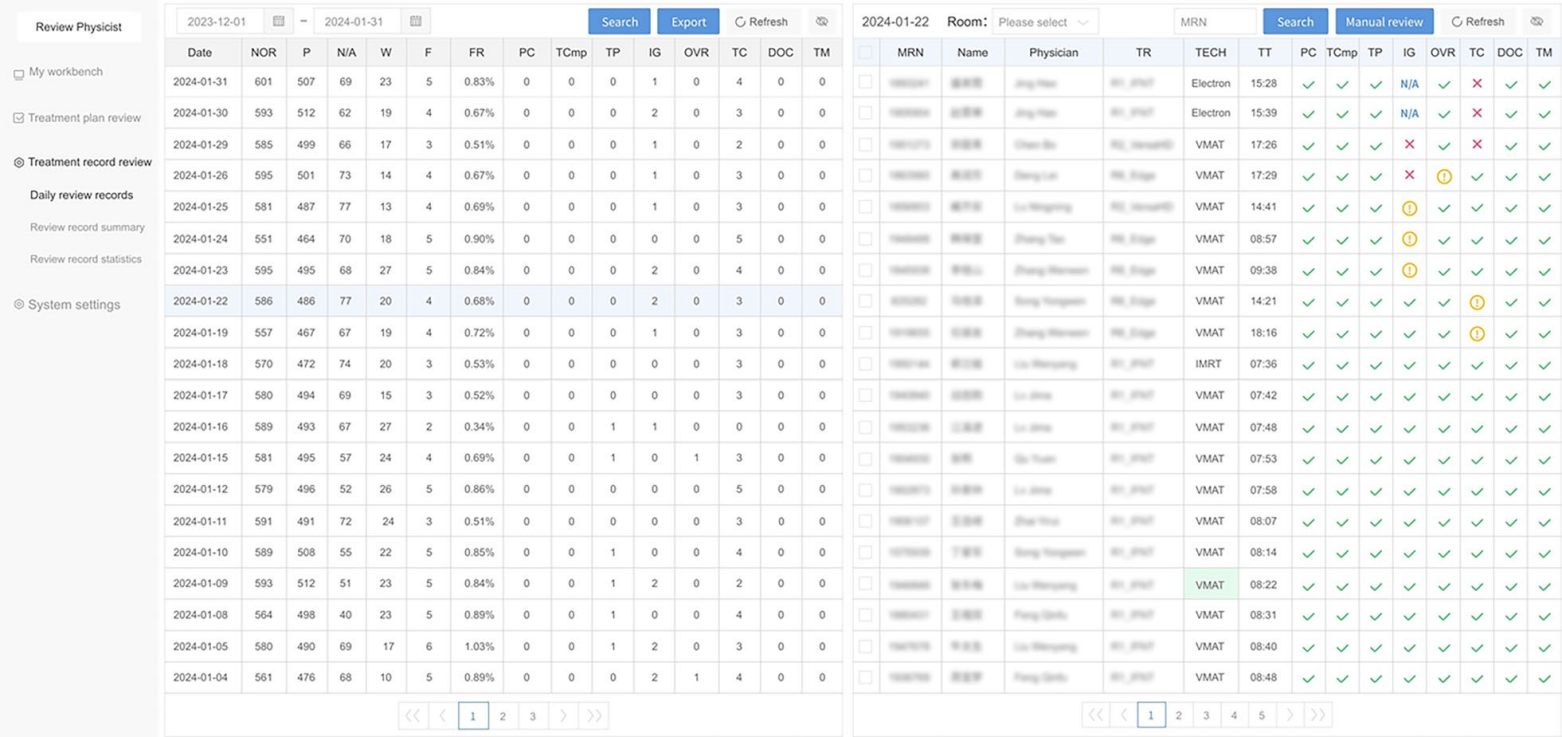
The review results provided by TRRS. NOR: Number of reviews, P: Pass, N/A: Not available, W: Warning, F: Failure, FR: Failure rate, PC: Parameter consistency, TCmp: Treatment completeness, TP: Treatment progression, IG: Image guidance, OVR: Override, TC: Treatment couch, DOC: Documentation, TM: Treatment mode, MRN: Medical record number, TR: Treatment room, TECH: Technique

TRRS automatically reviewed a total of 76,651 treatments from 4230 patients with an average of 574 treatment fractions per day. The percentage of the detected anomalies among the total records was 0.76%. The result of daily treatment records processed by TRRS is shown in Fig. 3. The list on the left is the summary of review results listed by date. The statistics include the total number of patients reviewed, the count of patients passing the review, the number of cases marked as N/A, the total instances of warnings, the overall count of failures, the failure rate, and the breakdown of failures across eight distinct review categories. The list on the right shows the overall review results of the selected day. The information include medical record number, patient name, physician, treatment room, technique, treatment time, and the review outcomes for the eight review categories.

The symbols are used to represent different review results. A "red cross" represents a failure. For instance, if the total monitor units (MU) of a patient's treatment fail to meet the planned value due to machine failure, the "completeness" item will display a red cross. An "yellow exclamation mark" represents a warning. For example, if a patient's treatment couch position deviation exceeds the specified lower limit but not the upper limit, the "couch position" item will display an orange exclamation mark. A "blue N/A" indicates that the review item is not applicable. For instance, in the case of electron beam therapy, IGRT checking is unnecessary, so the result will display blue N/A. A "Green check" represents a pass. If there are no abnormal values detected, the review item will display a green check.

**Fig. 4:**
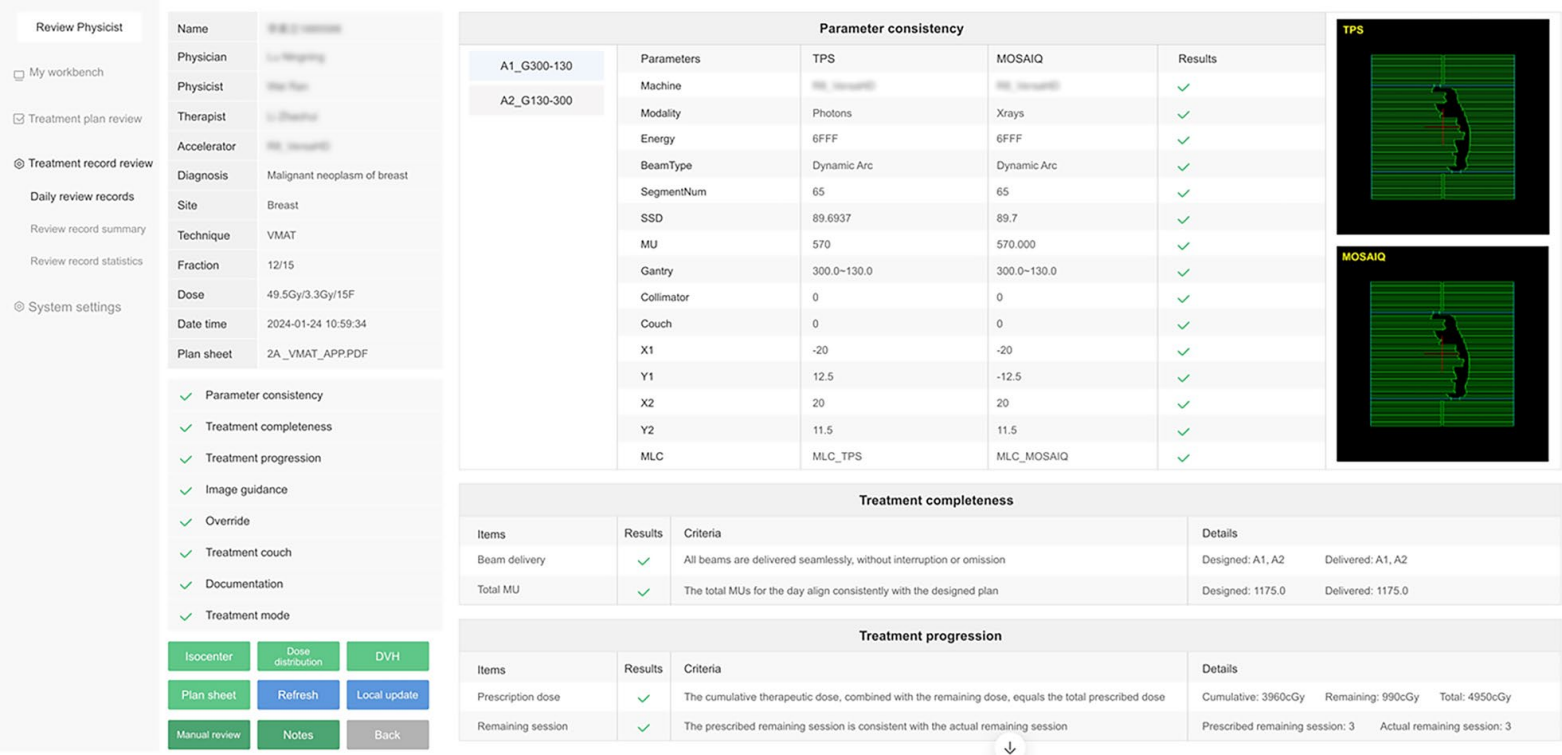
The review result of a daily treatment record provided by TRRS

The specific partial review results of a patient treatment records are shown in Fig. 4. The "parameter consistency" item checks whether the detailed parameters of the treatment fields consistent with the original plan. The "Treatment completeness" item checks whether all treatment fields have been conducted as planned without any unexpected interruptions. Additionally, it checks if the total MU administered during the treatment session consistent with the planned value.

The average processing time per treatment record is 3.9 s and 282 s for TRRS and manual process, respectively. Comparing with manual process, the time efficiency of TRRS is improved by a factor of 72. The average numbers of anomalies detected by the automatic and manual processes are 21 and 13 among total 631 daily records, respectively. TRRS detected 61.5% more anomalies than those of the manual process. The percentages of anomalies detected by automatic and manual processes are 3.33% and 2.06%. The difference between the percentages of anomalies detected by both processes is 1.27% which is not significant statistically. Note that the anomalies detected by manual process are also included in the anomalies detected by TRRS. These anomalies are mostly not incident and can be fixed beforehand. The actual incident happened but very rare (about 1–2 times per year).

## Discussion

The goal of radiotherapy is to deliver the treatment plan to patient positioned on the treatment couch. Thereby the planning dose distribution in patient body can be achieved. The treatment delivery consists of two steps: (1) Positioning patient on treatment couch using body fixation and immobilization devices, (2) Downloading and executing treatment plan on the treatment console. Both tasks highly rely on the proficiency of radiation therapists. Under normal condition of treatment machine, high delivery accuracy of treatment plan can be guaranteed. However, the daily repetitive and high-demanding clinical work is susceptible to human errors. Although the advanced treatment control system and record & verify (R&V) system are used to minimize the treatment errors, complete elimination of them is less possible. Huang et al. conducted an analysis for a period of 5 years and found 555 errors among 28,136 patient treatments (average 1.97 error per 100 patients) [2]. Bissonnette et al. analyzed 1063 incident reports from 2001 to 2007, revealing an average incident rate of 1.7 per 100 radiotherapy courses [3].

The AAPM TG 275 provides a comprehensive list of inspection items relevant to the review of treatment records. For patients undergoing treatment, a minimum weekly review of treatment records is recommended. For patients undergoing Stereotactic Radiosurgery (SRS) or Stereotactic Body Radiotherapy (SBRT), a more rigorous review frequency of treatment records is recommended to enhance quality assurance measures. Although the review of a treatment record (TRRS) includes fewer items than the review of a treatment plan (TPRS), the manual review of a treatment records still imposes a considerable work on the clinical physicists [13–18]. Alternatively, many institutions opt to simplify review items or extend review intervals to reduce the workload. The introduction of computer-aided systems relieve physicists from this repetitive work and let them focus on more valuable tasks such as checking those anomalous treatment records detected by TRRS.

Both TPRS and TRRS are QA procedures which are implemented in our department for clinical use. TRRS is an extension of TPRS. Upon completion of a radiotherapy plan, TRRS automatically checks relevant parameters to ensure plan delivery accuracy [22]. Previously, checking the consistency between the treatment plan and delivery was a challenging task. The implementation of TRRS perfectly solves this issue. During the treatment plan review process, TPRS extracts original plan files from TPS, converting them into structured data. It is then linked to the "site" table in the R&V system, allowing TRRS to accurately match the current treatment fraction to its corresponding original plan in TPRS using the "primary key" in the "site" table. Note that the current commercial R&V systems or the Treatment Management Systems (TMS) performs thorough consistency check between plan parameters during delivery and those store in the R&V system, which make it unnecessary to double checked by TRRS.

The review items in TRRS are formulated with reference to the checklist recommended by the AAPM TG275 and MPPG11.a. In addition, they also based on many years’ experience on daily treatment record review in our department. The system includes most of potential anomalous events during radiotherapy plan delivery. The review rules are carefully designed to address problems in various clinical scenarios. With the rigorous tests by review physicists, the system is expected to minimize the false negative rate to zero at our best efforts. While comparing with the checklists provided by TG275 and MPPG11.a, the majority of the recommended review items were implemented in our TRRS.

It is cautious to devise overly strict tolerances or criteria. Stricter rules may result in high false positive rate, leading to unnecessary errors or warning message, and misleading the review physicist's attention. For instance, after completing positioning verification for the first treatment fraction using CBCT, TMS performs a 6D correction of the treatment couch based on the registration results on Edge (Varian Medical System). Consequently, there is a substantial deviation between the couch position recorded by the R&V system and the preset position before treatment. This kind of deviations can be judged as normal or anomalous events according to the different clinical protocols. Therefore, review rules should be carefully devised to avoid high false positive results.

While the TRRS offers significant improvements in the efficiency and accuracy of treatment record reviews, there are several limitations. First, the system relies on rules/criteria to identify the potential issues. This may fail to detect certain anomalies in the complex and unanticipated clinical conditions or scenarios. Second, the data of TRRS was only obtained from the R&V system and the review results of the TPRS, this limit its capability to collect data from various clinical devices and database. Third, there are not available incident and anomaly databases established across the country. The improvement of TRRS is difficult without the input of the accumulated incident reports.

To ensure reliability, TRRS was tested on the patient treatment records over a 6-month period. TRRS was compared with manual review results to evaluate its accuracy in identifying anomalies. Additionally, a continuous monitoring and feedback mechanism was established. They include regular sampling of patient records for TRRS testing, comparing the results of TRRS results with those performed by human operators, deliberately introducing errors in records for fault-tolerance testing. These measures would further enhance and improve the system's reliability.

In alignment with medical device regulations, the TRRS was developed in accordance with international standards, particularly ISO 13485 for quality management systems and IEC 62304 for software development in medical devices. The system underwent rigorous verification and validation procedures, including functional testing, integration testing, and user acceptance testing. Throughout the development process, extensive documentation was maintained, covering aspects such as risk management and mitigation strategies. Additionally, regular audits and reviews are conducted to ensure that the system remains safe and effective for clinical use.

## Conclusion

TRRS improved the efficiency and effectiveness of reviewing process for daily patient treatment records of radiotherapy plans. The system not only extends the scope and frequency of review process but also promotes the detection rate of anomalies comparing to those of manual process. The implementation of TRRS can significantly relieve the workload of review physicists and enable them to focus on more important tasks related to the safety of patient treatment.

## Appendix 1

The detailed description of what rules and checks were implemented in this study, as follows:

**Table:** 

Categories	Record value/action	Reference value/action	Data type	Check rules	Tolerance
Parameter consistency	Machine	Machine in reviewed plan	String	Equal	N/A
	Modality	Modality in reviewed plan	String	Equal	N/A
	Energy	Energy in reviewed plan	Number	Equal	N/A
	Beam type	Beam type in reviewed plan	String	Equal	N/A
	Segment count	Segment count in reviewed plan	Number	Equal	N/A
	SSD	SSD in reviewed plan	Number	Within	0.1 cm
	MU	MU in reviewed plan	Number	Within	0.1
	Gantry angle	Gantry angle in reviewed plan	Number	Equal	N/A
	Collimator angle	Collimator in reviewed plan	Number	Equal	N/A
	Couch angle	Couch angle in reviewed plan	Number	Equal	N/A
	Jaw X1	Jaw X1 in reviewed plan	Number	Equal	N/A
	Jaw Y1	Jaw Y1 in reviewed plan	Number	Equal	N/A
	Jaw X2	Jaw X2 in reviewed plan	Number	Equal	N/A
	Jaw Y2	Jaw Y2 in reviewed plan	Number	Equal	N/A
	The position of each MLC leaf at the first control point	MLC leaf in reviewed plan	Array	Equal	N/A
Treatment completeness	MU delivered per field	MU delivered per field in reviewed plan	Number	Equal	N/A
	Total MUs	Total MUs in reviewed plan	Number	Equal	N/A
Treatment progression	Cumulative dose	Total prescribed dose minus remaining dose	Number	Equal	N/A
	Number of remaining treatment fractions in the prescription	Actual number of remaining treatment fractions	Number	Equal	N/A
	Daily treatment dose	Prescribed dose	Number	Equal	N/A
	Treatment calendar	Delaying or stopping treatment	Boolean Value	False	N/A
	Dose verification	Performed before stereotactic treatment	Boolean Value	True	N/A
Image guidance	Image status	Image approved	String	Equal	N/A
	Shifts applied	Couch position changes	Number	Equal	N/A
	Isocenter on the CBCT	Isocenter in reviewed plan	Number	Equal	N/A
	IGRT scan parameters	Match clinical requirements	Array	Equal	N/A
	IGRT frequency	Comply with MD directive	Number	Equal	N/A
	IGRT registration deviation	Predefined threshold	Number	Within	0.7–1.5 cm
Overrides	Couch position override	N/A	Boolean Value	False	N/A
	Field parameters override	N/A	Boolean Value	False	N/A
	Dose tracking override	N/A	Boolean Value	False	N/A
	Treatment fractionation mode override	N/A	Boolean Value	False	N/A
	Other overrides	N/A	Boolean Value	False	N/A
Treatment couch	Vertical treatment couch position	Vertical reference couch position	Number	Within	0.5–1.0 cm
	Lateral treatment couch position	Lateral reference couch position	Number	Within	0.5–1.0 cm
	Longitudinal treatment couch position	Longitudinal reference couch position	Number	Within	0.5–1.0 cm
Documentation	ID of each treatment-related documentation	Document ID	Number	True	N/A
	The staff who approve the documents	Planning physicist and review physicist	String	Equal	N/A
Treatment mode	Delivery mode	QA mode	Boolean Value	True	N/A
	Type of staff performing the QA model treatment	Physicist or engineer	String	Equal	N/A

Equal: The record value/action is equal to the reference value

Within: The record value/action is within the tolerance of the reference value

True: The record value/action is filled/performed

False: The record value/action is not filled/performed

## Data Availability

No datasets were generated or analysed during the current study.
